# Takayasu arteritis in children

**DOI:** 10.1186/1546-0096-6-17

**Published:** 2008-09-28

**Authors:** Safia Al abrawi, Marine Fouillet-Desjonqueres, Louis David, Xavier Barral, Pierre Cochat, Rolando Cimaz

**Affiliations:** 1 Département de pédiatrie, Hôpital Edouard-Herriot and Université Claude-Bernard Lyon1, Lyon, France; 2 Service de chirurgie vasculaire, CHU Saint-Etienne, France

## Abstract

Takayasu arteritis (TA) is a large vessel vasculitis that usually affects young female patients during the second and third decades of life, but has been reported in children as young as 24 months of age. Aim of this report was to describe four children (two girls) with TA, as well as summarizing main published studies. The mean age at presentation of our cases was 11 years (range 8–15). Three patients were Caucasians and one Asian. Arterial hypertension was the commonest mode of presentation followed by systemic symptoms. Other related symptoms were due to ischemia and consisted of abdomen, chest, and limb pain. An abdominal bruit was noted in only one patient. Inflammation markers were always abnormal. Angiography was performed in all cases; left subclavian artery and common carotid artery were more frequently involved. Renal artery stenosis was observed in two patients. One boy was diagnosed as having an associated immune deficiency (Wiskott-Aldrich syndrome). Treatment modalities included prednisone (n = 4), methotrexate (n = 3), and mycophenolate mofetil (MMF) (n = 1). Surgery was required in two patients. Follow-up ranged from 3 to 10 years since diagnosis. In three cases antihypertensive drugs and methotrexate were stopped, and prednisone was reduced to 7.5 mg/day.

## Introduction

Takayasu arteritis (TA) is a large vessel vasculitis affecting mainly the aorta and its major branches. TA occurs most commonly in female patients in the second and third decades of life, but has also been reported in children as young as 24 months of age [1]. The disease is more frequent in Asian populations, but has been reported in patients of all ethnical background. Descriptions of TA in the pediatric age are scanty. Moreover, treatment options have been limited so far, with few reports focusing on immunosuppressive treatment (2 methotrexate, 1 cyclophosphamide) in pediatric TA [2-4].

The aim of the present report was to describe four cases of pediatric TA seen in a French tertiary pediatric rheumatology center, with an emphasis on both medical and surgical management, as well as providing a recent review of the literature. A representative clinical history is detailed below, while Table 1 summarizes the characteristics of the patients.

**Table 1:** Characteristics of our patients with Takayasu arteritis.

	Patient 1	Patient 2	Patient 3	Patient 4
Age at diagnosis (years)	12	11	8	15
Gender (M, male; F, female)	F	M	F	M
Ethnicity	Caucasian	Asian	Caucasian	Caucasian
Clinical presentation	-pain left arm	-hypertension	- hypertension	-acute chest pain
	-absent radial and brachial pulse, weak left carotid pulse		-abdominal pain	
Laboratory findings at disease onset:				
-ESR (mm/hr)	80	125	100	80
-CRP (mg/l)	60	80	134	60
-Hb (g/l)	100	79	92	110
-platelets (G/l)	400	738	487	745
Treatment (initial dose):				
-prednisone (mg/kg/day)	2	2	2	1.5
-methotrexate (mg/m^2^/wk)	10	0	10	10
-MMF (mg bid)	0	250	0	0
Surgical intervention	none	Bypass brachiocephalic trunk	Bypass abdominal aorta	None
Follow up (years)	10	3	9	3
Outcome and treatment at last visit	-Clinical remission	- Clinical remission	- Clinical remission	- Clinical remission
	-prednisone 5 mg/d	-prednisone 7.5 mg/d MMF 750 mg bid	-aspirin 100 mg/d	-prednisone 7.5 mg/d

## Case presentation

**Figure 1:**
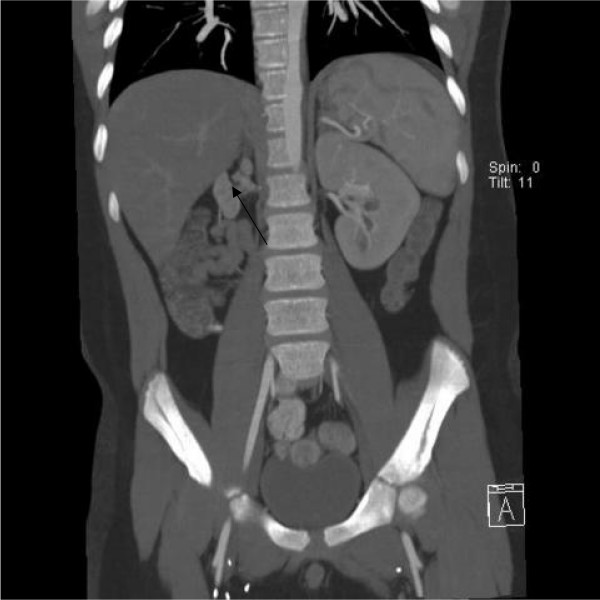
MRA showing right renal artery stenosis with small right kidney (arrow).

PH, male, with unremarkable family or past medical history, at the age of 11 years was admitted for the occurrence of arterial hypertension (180/100 mm Hg) with the presence of headache and vomiting for the previous 2 days. His parents reported a history of fatigue, myalgia, frontal headache and weight loss (9 kg) for the previous 2 months. On admission he was alert, afebrile and in no acute distress. BP was 170/105 mm Hg. All his pulses were felt and symmetric. Fundoscopy was normal. No abdominal bruit was present. The rest of his physical examination was normal. Investigations on admission revealed anemia (Hb 79 g/L), thrombocytosis (platelet count 738 G/L), ESR 125 mm/h, CRP 80 mg/L. The rest of laboratory investigations, including serum creatinine, electrolytes, autoantibodies, and urinalysis, were normal, as well as ECG and chest X-ray. An echocardiogram showed mild thickening of the left ventricle. A renal color Doppler ultrasound examination revealed a right renal artery stenosis with a small ipsilateral kidney (70 mm vs 104 mm for the left kidney). An angio-MRI demonstrated a thrombosis in the left common carotid artery, a 70% stenosis of the left subclavian artery, a 80% stenosis of the brachiocephalic artery, a thrombosis in the descending thoracic aorta, and a right renal artery thrombosis and stenosis (Figure 1). The boy was given prednisone 2 mg/kg per day, antihypertensives (amlodipine 5 mg/day, enalapril 10 mg/day), as well as low-dose aspirin. After 2 weeks, ESR fell to 26 mm/h and BP to 156/68 mm Hg. Surgery was planned, but the patient relapsed at the prednisone dose of 5 mg per day, so that daily dosage was increased up to 40 mg (1 mg/kg/d), and another immunosuppressive (mycophenolate mofetil, MMF, 250 mg bid) was added. Six months later he underwent revascularization bypass surgery of brachiocephalic trunk (a femoral vein was used as a graft between ascending aorta and right carotid artery). He was discharged two weeks after surgery on prednisone 10 mg/day, MMF 250 mg bid, 4 antihypertensive drugs (enalapril 10 mg/day, nicardipine 60 mg/day, labetalol 400 mg/day, and clonidine 0.3 mg/day) and low-dose aspirin. At last visit at 14 years of age, BP was controlled (135/65 mmHg) by three medications; he was taking prednisone 7.5 mg per day, MMF (750 mg bid), and low-dose aspirin. His last investigation showed ESR 28 mm/h, Hb 115 g/dL, platelet count 557 G/L, and a normal renal function. His last MRI angiogram showed normal visualization of the brachiocephalic trunk and right internal carotid artery. Neurological examination was normal, but periodic headaches were still present.

## Discussion

Takayasu arteritis has been rarely reported in childhood. A common clinical mode of disease presentation in our patients was arterial hypertension (2/4 cases), together with nonspecific symptoms (headache, fatigue, myalgia, weight loss). Symptoms due to ischemia, which are frequent in adults, have been seldom reported in children. However, although true claudication (upon effort) was present only in one patient, we have observed ischemic findings (chest, limb, and abdominal pain) in three of our four patients. The disease is also called 'pulseless disease', since peripheral pulses are often absent due to vascular obstruction; however, this feature was present only in one of our patients. In addition, an abdominal bruit can often help in the diagnosis, but this was noted in only 1/4 of our cases.

The largest series of TA in children has been reported by Hong et al., who described 70 cases [5]; the male to female ratio was 1:4.4 and the youngest patient was 3 years old. Arterial hypertension was seen in 65/70 patients (93%). In another report of 31 children with TA from South Africa [6], arterial hypertension was the most common presenting feature, followed by cardiac failure, bruits, and absent pulses. Jain et al [7] from India reported 24 children with TA; again, arterial hypertension was the commonest mode of presentation, seen in 83% of patients; the male to female ratio was 1:5. In a Turkish multicenter serie, TA represented 1.5% of pediatric vasculitides [8]. Among these patients, 71.4% described constitutional symptoms. Hypertension was the leading feature, and renal involvement was present in 86% of cases. Half of the patients had involvement of both thoracic and abdominal aorta. Table 2 summarizes clinical findings of main published series, as well as our own data.

**Table 2:** Summary of main clinical features in our series and in published reports of pediatric Takayasu arteritis.

	Gender F/M	Hypertension	Elevated inflammatory parameters	Claudication	Renal artery involvement	Abdominal aortic involvement	Thoracic aortic involvement
Present series	2/2	50%	100%	25%	25%	25%	25%
Ozen et al. [8]	7/7	86%	100%	NA	NA	57.1%	28.5%
Hong et al. [5]	57/13	93%	NA	NA	NA	NA	NA
Hahn et al. [6]	13/18	84%	74%	13%	71%	42%	16%
Jain et al. [7]	4/20	83%	42%	NA	75%	71%	21%

NA, data not available.

The diagnosis of TA is based on characteristic findings of diseased aorta and its major branches seen on angiography. This is demonstrated by luminal abnormalities such as stenosis or aneurysmal dilatation of the aorta, its major branches, and the pulmonary arteries. With regard to imaging studies, traditionally the angiographic patterns have been divided in: type I, affecting the aortic arch; type II, the thoracic and abdominal aorta; type III, the aorta both above and below the diaphragm; and type IV, the aorta and the pulmonary arteries. In our cases type I was the predominant pattern, whereas in two series type II was the predominant one [7,9].

Ultrasonography and positron emission tomography are new, promising techniques to assess large-vessel vasculitides. Color-coded Doppler sonography can facilitate an accurate diagnosis of Takayasu arteritis by the characteristic appearance. Homogeneous circumferential intima-media thickening of the common carotid arteries is a specific ultrasonographic finding in patients with Takayasu arteritis [10-12]. More recently MRI has been used to establish the diagnosis of TA in children, to monitor disease activity and to guide treatment. Early in the disease of TA, smooth muscle thickened vessel walls, which may be the only manifestation of vascular inflammation, may be not detected by conventional angiography but MRI can visualize the thickened vessel wall directly, and in addition it can show other signs of active inflammation such as mural edema with T2-weighted imaging and increased wall vascularity with enhanced imaging [13,14]. For one patient in our series MRI was used to establish the diagnosis of TA and for follow-up. Table 3 summarizes the evolution of vascular involvement in our patients, as studied by echodoppler, MRI and/or angiography.

**Table 3:** Vascular involvement during the disease course of our patients as investigated by imaging studies.

	At diagnosis	On treatment (2–5 years after diagnosis)	During a disease flare	At last visit
Patient 1	**Echodoppler:** thrombosis left subclavian artery, left carotid thickening	**Echodoppler:** stability of previous findings	**Echodoppler**: After 6 years thickening of left carotid artery	**Echodoppler:** stability of left carotid thickening
		**Angiography** -reduction left subclavian artery diameter without thrombosis, hypoplastic left carotid without parietal lesions		
Patient 2	**angioMRI: **thrombosis left common carotid artery, stenosis left subclavian artery, stenosis of the brachiocephalic artery, thrombosis descending thoracic aorta, right renal artery thrombosis and stenosis.	**Echodoppler: (after surgery):** patency of left carotid and carotid trunk	No flares	**angioMRI:** normal visualization of the brachiocephalic trunk and right internal carotid artery
	**Echodoppler:** left carotid thrombosis, left subclavian stenosis, tronc brachiocephalic trunk stenosis			
Patient 3	**Angiography:** left subclavian stenosis, abdominal aortic stenosis, superior mesenteric artery stenosis, bilateral renal artery stenosis	**Echodoppler:** stability subclavian lesion, improvement aortic wall involvement	**Echodoppler:** stability of subclavian and aortic lesions	**Angiography:** stenosis abdominal aorta
Patient 4	**Echodoppler:** left subclavian thrombosis			

With regard to treatment, in our series corticosteroids have been used in all cases, with the adjunction of methotrexate in three cases and MMF in one. Although the numbers are small and the retrospective nature of this report does not allow to draw firm conclusions, our patients have obtained clinical and laboratory remission with this regimen of early immunosuppression. Our patients were followed from 2 to 10 years since diagnosis (mean, 6 years) and medications at the last visit included only low-dose prednisone in three cases (one of whom still needed MMF and anti-hypertensives), while the fourth patient is now only on low-dose aspirin. Bypass surgery was required in two patients because of severe vascular occlusion, with excellent results as well. One bypass was carried out between the ascending aorta and the right carotid artery, the other one between the lower thoracic aorta and infra-renal aorta; in the latter a reimplantation of left renal artery and auto transplantation of right renal artery to common iliac was also performed.

Due to the rarity of the disease, there are no controlled studies of medical treatment of children with TA. An interesting new possibility is represented by the use of sildenafil, that has been recently reported in a 8-year old girl [15]. We have been able to find in the literature only a previous French case, a 6 year-old girl who was treated with methotrexate [4]. Methotrexate has been used both in adults and children with good results [16,17]. Mycophenolate mofetil (MMF) has also recently been introduced in the treatment of adult patients with TA [18].

TA has been associated with other autoimmune diseases such as systemic lupus erythematosus, juvenile idiopathic arthritis, anterior uveitis, sarcoidosis, seronegative spondyloarthropathy, Crohn's disease, Wegener's granulomatosis, and Sweet syndrome [19-26]. One boy in our series, who suffered from eczema and thrombocytopenia since infancy, was diagnosed as having Wiskott-Aldrich syndrome (WAS) at the age of 16 years; this was subsequently confirmed by genetic study. The peculiar association of TA and WAS has already been mentioned in another previous report [27].

TA is a disease with severe prognosis, mortality rate being reported in children from 35 to 40% by five years [28]. It is therefore important to have a high index of suspicion and in doubtful cases a low threshold for diagnostic evaluation. We underline the possibility of TA in any young patient with unexplained arterial hypertension.

We conclude that TA is not such a rare disease in a pediatric rheumatology setting, and also that it has to be considered in cases of unexplained arterial hypertension or unexplained inflammatory syndromes without signs of localization. A thorough physical examination can lead to the correct diagnosis if pulses cannot be felt or if an abdominal bruit is heard, even if these are not constant findings. Since the disease can be progressive and life-threatening, an early recognition is vital in order to start immunosuppression, which proved to be very successful in our patients.

## Competing interests

The authors declare that they have no competing interests.

## Authors' contributions

SAA and RC wrote the manuscript, MFD and LD retrieved data from charts, and XB and PC conceived and supervised the study. All authors read and approved the final manuscript.

## References

[B1] Ladhani S, Tulloh R, Anderson D (2001). Takayasu disease masquerading as interruption aorta in a 2 years old child. Cardiol Young.

[B2] Shetty AK, Stopa AR, Gedalia A (1998). Low dose methotrexate as a steroid-sparing agent in a child with Takayasu arteritis. Clin Exp Rheumatol.

[B3] Brunette MG, Bonny Y, Spigelbatt L, Barrette G (1996). Long term immunosuppressive treatment of a child with Takayasu arteritis and high IgE immunoglobulins. Pediatr Nephrol.

[B4] Besson-Léaud L, Grenier N, Besson-Léaud M, Boniface C, Guillard JM (2001). Maladie de Takayasu: intérêt du traitement par méthotrexate. Arch Pediatr.

[B5] Hong CY, Yung YS, Choi JY, Sul JH, Lee KS, Cha SH (1992). Takayasu arteritis in Korean children clinical report of seventy cases. Heart Vessels.

[B6] Hahn D, Thomson PD, Kala U, Beale PG, Levin SE (1998). A review of Takayasu arteritis in children in Gauteng, South Africa. Pediatr Nephrol.

[B7] Jain S, Sharma N, Singh S, Bali HK, Kumar L, Sharma BK (2000). Takayasu arteritis in children and young Indians. Int J Cardiol.

[B8] Ozen S, Bakkaloglu A, Dusunsel R, Soylemezoglu O, Ozaltin F, Poyrazoglu H, Kasapcopur O, Ozkaya O, Yalcinkaya F, Balat A, Kural N, Donmez O, Alpay H, Anarat A, Mir S, Gur-Guven A, Sonmez F, Gok F, Turkish Pediatric Vasculitis Study Group (2007). Childhood vasculitides in Turkey: a nationwide survey. Clin Rheumatol.

[B9] Muranjan MN, Bavdekar SB, More V, Deshmukh H, Tripathi M, Vaswani R (2000). Study of Takayasu arteritis in children: clinical profile and management. J Postgrad Med.

[B10] Schmidt WA, Blackmans D (2005). Use of ultrasonography and position emission tomography in the diagnosis and assessment of large-vessel vasculitis. Curr Opin Rheumatol.

[B11] Schmidt WA, Nerenheim A, Seipelt E, Poehls C, Gromnica-Ihle E (2002). Diagnosis of early Takayasu artertis with sonography. Rheumatology (Oxford).

[B12] Chaubal N, Dighe M, Shah M (2004). Sonographic and color Doppler finding in aortoarteritis Takayasu arteritis. J Ultrasound Med.

[B13] Aluquin VP, Albano SA, Chan F, Sandborg C, Pitlick PT (2002). Magnetic resonance imaging in the diagnosis and follow up of Takayasu artertis in children. Ann Rheum Dis.

[B14] Choe YH, Han BK, Koh EM, Kim DK, Do YS, Lee WR (2000). Takayasu arteritis: assessment of disease activity with contrast- enhanced MR imaging. AJR Am J Roentgenol.

[B15] Uthman IW, Chaaban H (2006). The use of sildenafil in pediatric Takayasu arteritis. Clin Rheumatol.

[B16] Hoffman GS, Leavitt RY, Kerr GS, Rotten M, Sneller MC, Fauci AS (1994). Treatment of glucocorticoid-resistant or relapsing Takayasu arteritis with methotrexate. Arthritis Rheum.

[B17] Sato EL, Lima DN, Espirito Santo B, Hata F (2000). Takayasu arteritis, treatment and prognosis in a university center in Brazil. Int J Cardiol.

[B18] Diana E, Schieppati A, Remuzzi G (1999). Mycophenolate Mofetil for the treatment of Takayasu arteritis: report of three cases. Ann Intern Med.

[B19] Hall S, Nelson AM (1986). Takayasu arteritis and juvenile rheumatoid arthritis. J Rheumatol.

[B20] Mc Donald MA, Ojaimi E, Favilla I (2004). Anterior uveitis in a child with Takayasu arteritis. Clin Exp Ophthalmol.

[B21] Opastirakul S, Chartapisak W, Sirivanichai C (2004). A girl with Takayasu arteritis with possible systemic lupus erythomatosus. Pediatr Nephrol.

[B22] Acar B, Yalcinkaya F, Ozturk B, Yuksel S, Ozcakar ZB, Fitoz S (2005). Seronegative spondyloarthropathy associated with Takayasu arteritis in a child. Clin Exp Rheumatol.

[B23] Van Elburg RM, Henar EL, Bijleveld CM, Prins TR, Heymans HS (1992). Vascular compromise prior to intestinal manifestations of Crohn disease in a 14 years old girl. J Pediatr Gastroenterol Nutr.

[B24] Rose CD, Eichenfield AH, Goldsmith DP, Athreya BH (1990). Early onset sarcoidosis with aortitis juvenile systemic granulomatosis. J Rheumatol.

[B25] Mejia-Hermandez C, Alvarez-Mendoza A, Deleon-Bojorge B (1999). Takayasu arteritis coexisting with Wegener granulomatosis in a teenager with renal insufficiency. Pediatr Dev Pathol.

[B26] Campos LMA, Castellanos ALZ, Afiune JY, Kiss MHB, Silva CAA (2005). Takayasu arteritis with aortic aneurysm associated with Sweet syndrome in childhood. Ann Rheum Dis.

[B27] Lau YL, Wong SN, Lawton WM (1992). Takayasu arteritis associated with Wiskott-Aldrich syndrome. J Paediatr Child Health.

[B28] Morale E, Pineda C, Martinez-Lavin M (1991). Takayasu arteritis children. J Rheumatol.

